# Pesticide exposure in Sri Lanka

**DOI:** 10.1093/ije/dyv369

**Published:** 2016-04-18

**Authors:** DW Knipe

**Affiliations:** ^1^ School of Social and Community Medicine, Canynge Hall, 39 Whatley Road, Bristol BS8 2PS, UK. E-mail: dee.knipe@bristol.ac.uk; ^2^ South Asian Clinical Toxicology Research Collaboration (SACTRC), Faculty of Medicine, University of Peradeniya, Peradeniya, Sri Lanka

Pesticides are commonly used worldwide both in agricultural and domestic settings. The benefits to society and agriculture are well documented. There are, however, dangers associated with the use of pesticides. Some pesticides, such as organophosphates (the most commonly used pesticide worldwide), kill pests through a biological pathway that may also cause harm to humans. Acute exposure to pesticides has been associated with negative health effects such as cancer, respiratory disease, Parkinson’s disease and death. ^1–4^ People in low- and middle-income countries (LMICs), such as Sri Lanka, often experience acute exposure alongside chronic exposure due to the widespread and largely unregulated use of pesticides. ^5^ The level of chronic pesticide exposure in LMICs is likely to be higher than in high-income countries, as a large proportion of rural families in Sri Lanka live close to agricultural fields and are not only exposed to pesticides when the fields are being sprayed ( Figures 1 and 2 ) but also have sustained exposure through their food and drink and through air particles. In addition, villagers in rural Sri Lanka often use the irrigation canals, rivers and lakes as a free source of bathing water ( Figure 3 ). This water source is likely to be heavily contaminated with pesticides, not only due to run-off but also as a direct consequence of the practices of pesticide sprayers ( Figure 4 ). ^6^

**Figure 1.**
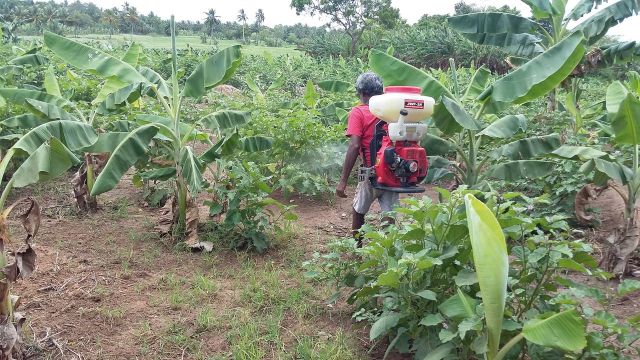
Pesticide spraying of banana crop using a power sprayer. No personal protection worn.

**Figure 2.**
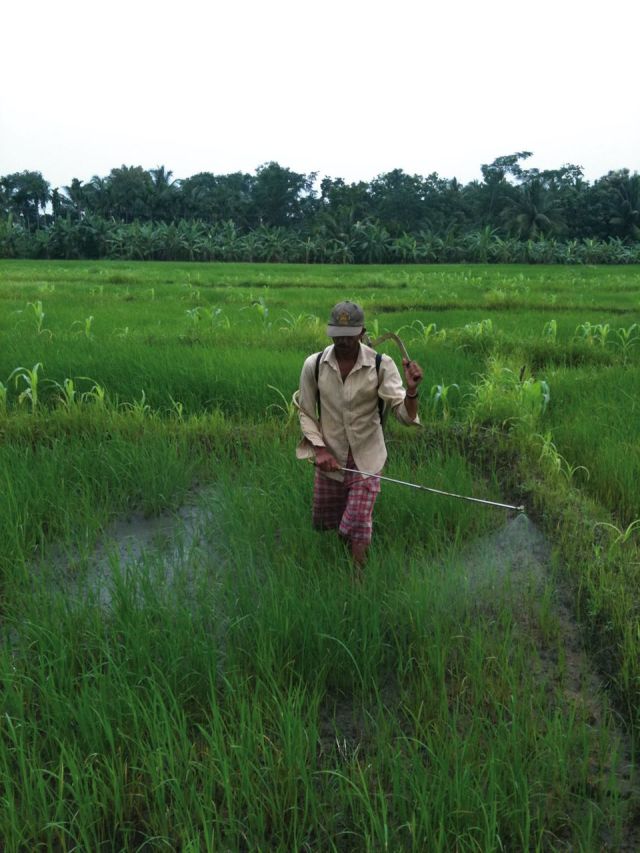
Manual pesticide spraying of a paddy crop. No personal protection worn.

**Figure 3.**
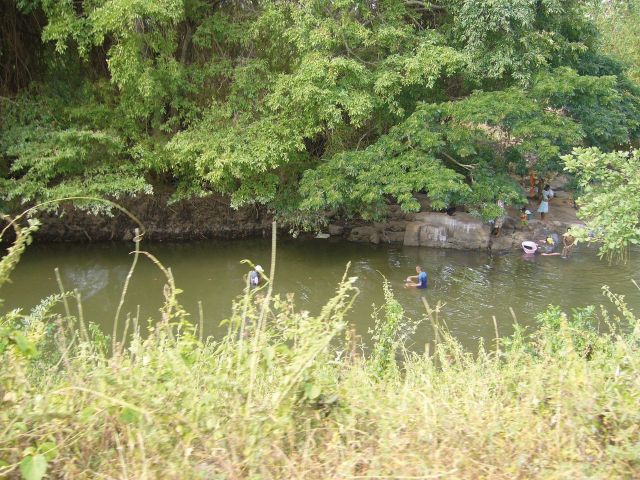
Villagers bathing and washing clothes in the local river.

**Figure 4.**
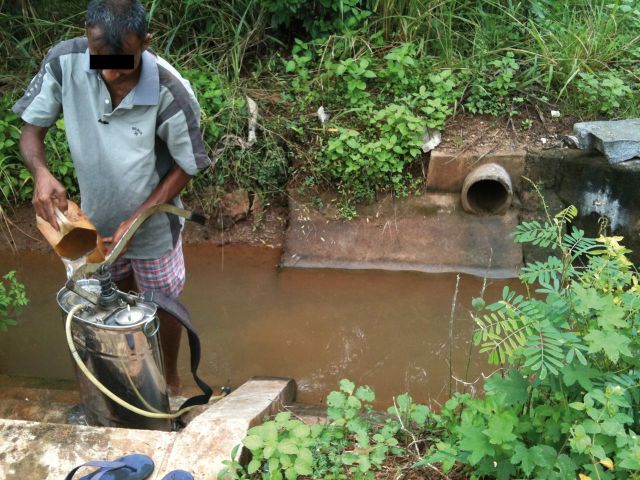
Farmer cleaning and preparing pesticide spraying equipment using the irrigation canal. Equipment is rinsed and water taken from the canal.

The success of a crop is imperative for a farmer’s livelihood in rural Sri Lanka. This need, coupled with heavy marketing of pesticides ( Figure 5 ) has led to a nearly ubiquitous use of pesticides on crops. ^7^ It is not clear whether this heavy use, resulting in high levels of both acute and chronic exposure, has any long-term detrimental health effects. The difficulty in assessing this arises because of challenges in the measurement of long-term pesticide exposure. Traditional biological samples, like blood and urine, are difficult to collect and only capture short-term pesticide exposure. ^8^

**Figure 5.**
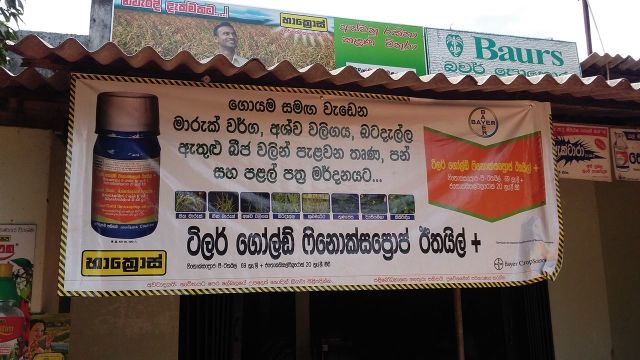
Example of a pesticide advertisment (approximate size 1 x 2.5 m).

In July 2015 a feasibility study was launched to determine whether it would be possible to measure chronic (long-term) exposure to pesticides using hair samples in rural Sri Lanka, as an alternative to blood/urine samples which are logistically difficult to manage in community settings in rural Asia. The additional benefit of hair samples is that pesticide metabolites deposit over time, and it would therefore be possible to ascertain a timeline of exposure over longer periods. ^9^ Following a village introduction ( Figure 6 ), fieldworkers visited households and recruited individuals to take part in the study. A hair sample (head/chest/forearm) was taken alongside self-reported information on pesticide use and exposure ( Figures 7 and 8 ). The samples will be analysed for organophosphate metabolites using gas chromatography-mass spectrometry ( Figure 9 ). Given that the knowledge of the long-term effects of the levels of pesticide exposure experienced in these countries is limited, there needs to be more research in this area to help inform legislation and effective community programmes for the safer use of pesticides.

**Figure 6.**
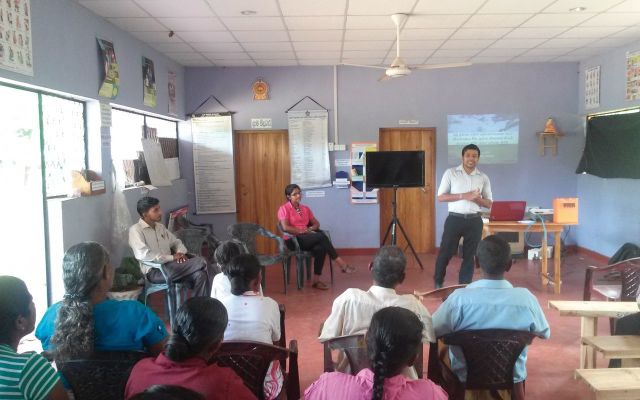
Village meeting to introduce the research project prior to home visits.

**Figure 7.**
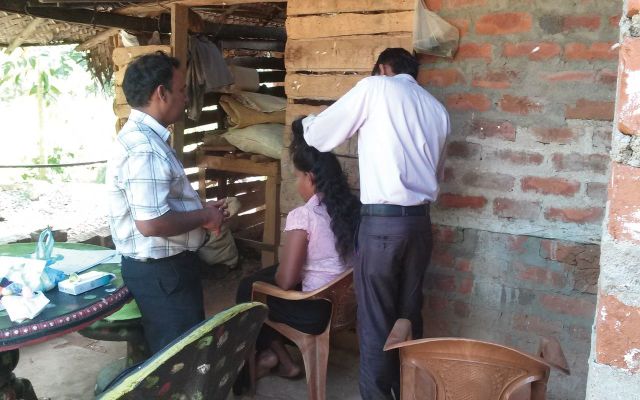
Field staff collecting head hair sample.

**Figure 8.**
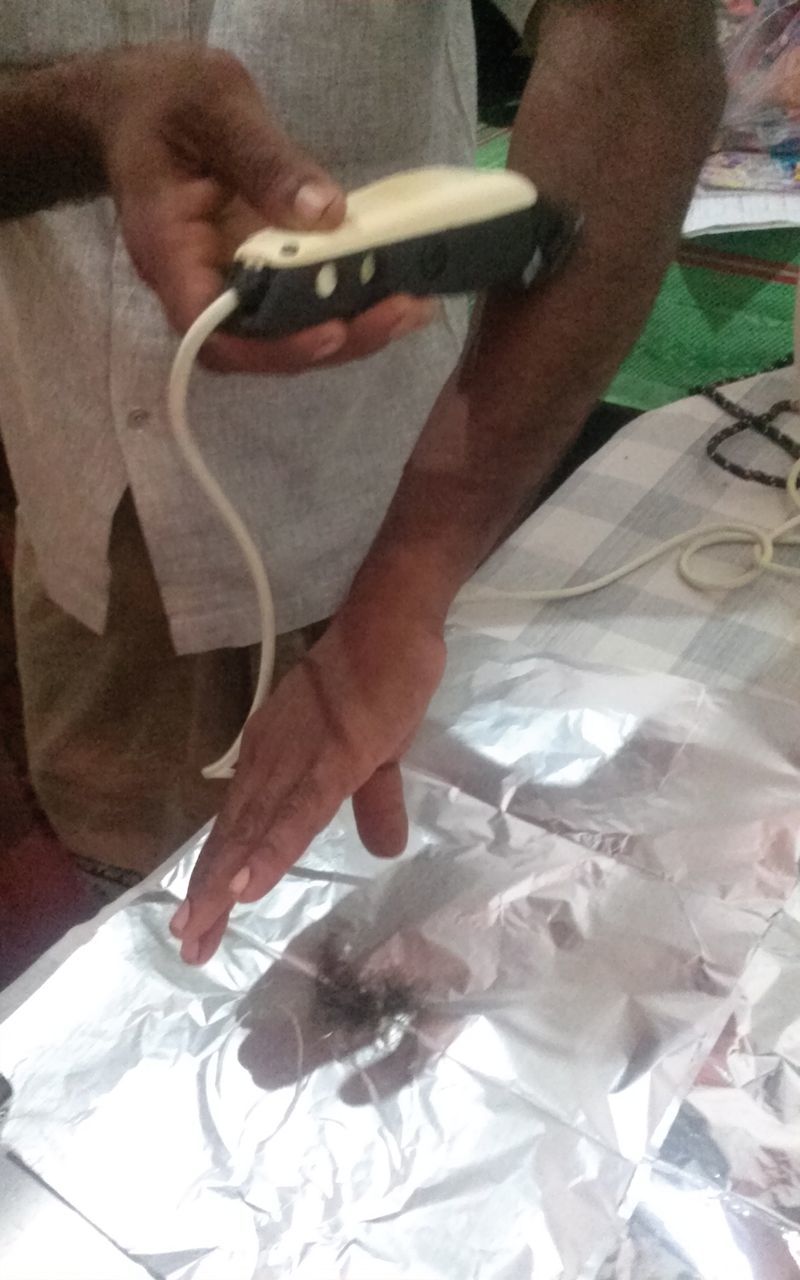
Alternative hair sample collected from men with limited or no head hair.

**Figure 9.**
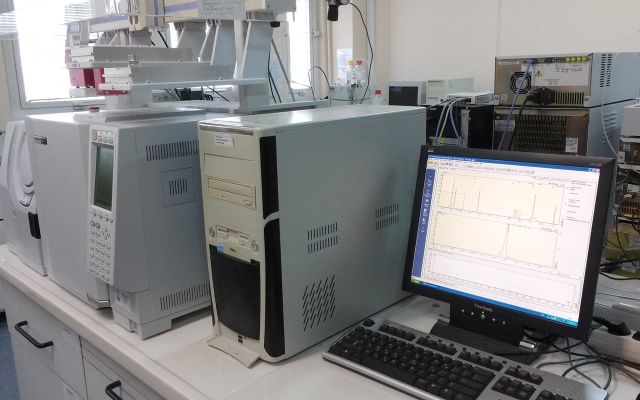
An example of the analysis output (pictured on the right) from organophosphate metabolite hair analysis using a gas chromatography-mass spectrometry (pictured on the left).

## Consent

The people (identifiable) photographed have given their consent for their pictures to be used in the dissemination and publication of this research.

## Funding

This work was supported by the Wellcome Trust [WT099874MA].

**Conflict of interest:** None declared.
